# Sex-Specific Factors Affecting Quality of Life After Major Trauma: Results of a Prospective Multicenter Registry-Based Cohort Study

**DOI:** 10.3390/healthcare13040437

**Published:** 2025-02-18

**Authors:** Viola Freigang, Karolina Müller, Antonio Ernstberger, Volker Alt, Anne Herrmann-Johns, Florian Baumann

**Affiliations:** 1 Department of Trauma, Regensburg University Medical Center, 93053 Regensburg, Germanyvolker.alt@ukr.de (V.A.); 2 Department for Epidemiology and Preventive Medicine, Division of Medical Sociology, University of Regensburg, 93051 Regensburg, Germany; anne.herrmann@klinik.uni-regensburg.de; 3 Faculty of Interdisciplinary Studies, Landshut University of Applied Sciences, Am Lurzenhof 1, 84036 Landshut, Germany; 4 Center for Clinical Studies, Regensburg University Medical Center, 93053 Regensburg, Germany; karolina.mueller@ukr.de

**Keywords:** health service research, management of major trauma, gender medicine, women’s health, outcome research, patient-reported outcome, HRQoL

## Abstract

**Background**: Major trauma is a leading cause of severe disability and mortality. The influence of patient sex on outcome after severe trauma is a topic of ongoing discussion. We present a prospective multicenter study on the effects of trauma severity on health-related quality of life (HRQoL) of female patients. We hypothesized that the impairment of HRQoL after major trauma between the sexes depends not only on sex itself but also on age. **Methods**: This multicenter prospective registry-based observational study compared sex-based differences in HRQoL of patients who sustained major trauma Injury Severity Score (ISS ≥ 16). The HRQoL was assessed using the EQ-5D-3L (European Quality of Life 5-Dimension 3-Level Version) score over 2 years post-trauma. **Results**: We included 416 patients (116 female/300 male) with an ISS > 16 (median ISS 22 IQR 18/30). All patients had a lower HRQoL after trauma than the population norm. Increased AIS (Abbreviated Injury Scale) face and extremity scores and ASA (American Society of Anesthesiologists) scores showed a significant decrease in HRQoL. Even though the groups of female and male patients were comparable in injury severity, female patients reported significantly more problems on the anxiety and depression scales than male patients 6 months (*p* = 0.003) and 24 months (*p* = 0.044) after trauma (6 months: female 46% vs. male 30%; 24 months: female 44% vs. male 32%). We observed the greatest improvement in the EQ Index over time in patients between 16 and 39 years of age, especially female patients (0.78 to 0.87 in females under 39 years of age, compared to males in the same age group 0.76 to 0.81). Females over 65 years of age initially presented the lowest EQ Index of 0.62. It remained significantly lower over time and was lower compared to male patients of the same age group (female EQ Index after 24 months was 0.68 compared to men over the age of 65 who presented an EQ Index of 0.75). **Conclusions**: All patients included in this study presented a lower HRQoL after trauma than the population norm. Female patients under 39 years of age reported the most improvement. Females over 65 years of age showed a limited HRQoL, which remained significantly lower over time. Female patients reported significantly more anxiety and depression after major trauma than male patients. Thus, further development and methodologically rigorous testing of ortho-geriatric initiatives, psychosocial support, and prevention measures are required to improve the care after major trauma, particularly for the female elderly.

## 1. Introduction

Evaluations of health-related quality of life (HRQoL) have gained importance as patient-centered outcome measures become more prominent in trauma research [1]. Patient-centered analysis methods like the multidimensional EQ-5D (European Quality of Life 5 Dimensions) score are more demanding than investigating mortality rates due to the longitudinal follow-up. However, given the severity of the personal consequences of major trauma, investigating the health-related quality of life (HRQoL) is fundamental [2,3].

Sex- and age-related differences in trauma response, injury patterns, and outcomes led to an increased recognition of independent risk factors for morbidity and mortality after major trauma. Recent publications have highlighted the role of biological and socio-cultural factors in shaping these disparities leading to a potential underestimation of female trauma patients [4]. Sex-specific hormonal influences on immune response may influence outcomes after trauma. Experimental evidence suggests that androgens contribute to immune depression in males following trauma hemorrhage, while female hormones may exert protective effects with estradiol-dependent hypercoagulopathy leading to less hemorrhage after trauma [5,6]. Despite these findings, clinical studies have produced conflicting results, with some indicating that the male sex is an independent predictor of morbidity, while others report no survival advantage for females [7,8]. Trauma leads to an increased production of cytokines, leading to an inflammatory response and suppression of the immune system [9]. Since the immune cells can produce sex steroids, an influence of sex on trauma response is likely [10]. Androgens cause immunodepression following trauma hemorrhage in males [11]. Other studies provided evidence of the protective nature of female hormones regarding sepsis, hemorrhage, and major trauma [5,7,8,12]. Despite substantial experimental evidence for sex-related outcome differences after major trauma, clinical studies have shown conflicting results [7,13].

Mortality was shown to be largely influenced by age and the Injury Severity Score (ISS), and sex appeared to be of greater influence in older age and high-impact trauma with a high ISS [14,15]. After severe trauma, geriatric patients may not regain functional ability and HRQoL [16]. Sex-based differences in access to trauma care have been identified, leading to a lesser likelihood of females being admitted to trauma center care and an ICU, causing questions about a subconscious sex bias [8,17]. There is evidence that female patients have a longer stay in the emergency department, have a higher rate of delay for definitive fixation of femur and pelvic fractures, and are more likely to be discharged to a long-term care facility than male patients [18,19,20,21]. In addition to disparities in care, patient age seems to play an important role in outcome after severe trauma [7,8,12,14,15,18,20].

The question remains if these disparities have an impact on HRQoL after major trauma. The purpose of this study was to shine a light on the association between sex and quality of life after major musculoskeletal trauma. We hypothesized that the impairment of HRQoL after major trauma between the sexes depends not only on sex itself but also on patient age.

## 2. Methods

Within the East Bavarian trauma network (TNO) [22], we conducted a multicenter prospective registry-based observational cohort study of all adult patients (≥16 years of age) with an ISS ≥ 16 and monitored the development of health-related quality of life over 24 months [22]. The German Federal Ministry of Education and Research granted funding for the study (reference number 01GY1153), and the University of Regensburg’s Ethics Committee approved it (reference number 10-101-0077). The study was registered in the German Clinical Study Register (reference number DRKS00010039) and the German Network of Health Services Research database (reference number VfD_Polyqualy_12_001978).

Five hundred and eight patients were interviewed, and their EQ-5D data were collected. Sex was recorded as self-reported sex assigned at birth. There were no non-binary participants or any discrepancy between anatomical attributes and reported gender identity.

Out of 2596 patients who were treated for severe trauma over two years, 56% of patients (n = 1453 patients) were excluded with an Injury Severity Score (ISS) below 16.

**Figure 1:**
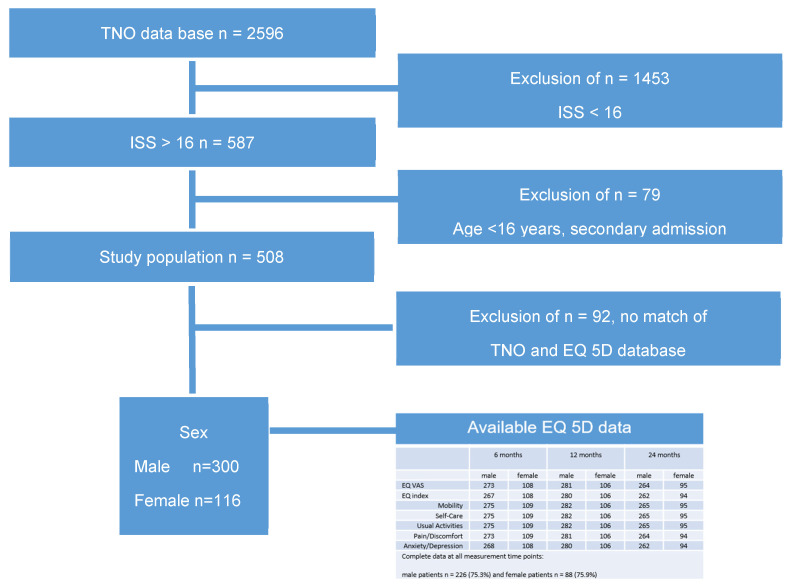
Selection procedure of the study population and EQ-5D data.

We were able to match TNO data and EQ-5D for 456 patients. After the exclusion of all patients below the age of 16 and all secondary admissions, 416 patients aged ≥ 16, ISS ≥ 16, and an HRQoL assessment were included in the present study (Figure 1).

We compared the baseline data of enrolled patients with patients lost to follow-up to maintain a representative sample and assess bias. No clinically meaningful variations existed between the excluded patient population and the participants.

We used the German Trauma Society’s (DGU) online registry to collect sociodemographic information and clinical data.

We evaluated the overall HRQoL at six, twelve, and twenty-four months following trauma, using the EQ-5D-3L. The EQ assesses the state of health concerning mobility, self-care, routine activities, pain/discomfort, and anxiety/depression. The replies were split into two categories: difficulties and no problems.

Furthermore, an EQ Index ranging from −0.21 (worst) to 1.00 (health) was created using five dimensions [23].

A 100 mm global health visual analog scale (EQ VAS), with 0 points representing the worst health and 100 representing the best health, is used in the second section of the EQ-5D-3L to gauge the patient’s assessment of their current global health status. The certified EQ-5D is offered in German [24,25].

Based on the Abbreviated Injury Scale, the ISS evaluates the degree of trauma (AIS). All three of the most affected body regions’ injuries are rated. The ISS is calculated by summing the squares of each score. An ISS of at least 16 is considered a major trauma [26].

A prognostic score used in the initial assessment of trauma patients is the RISC II. It consists of ten clinical and laboratory parameters to assess early mortality after severe trauma [27].

The patient’s functional impairment is estimated using the functional capacity index (FCI). Ten physical functions serve as its foundation, and these are assessed and converted into a numerical score in a range of 0 to 100. When the index is 100, there is no functional restriction [28].

## 3. Statistics

SPSS (Version 29, SPSS Inc., Chicago, IL, USA) was used for statistical analysis. For every test, the significance level was established at *p* ≤ 0.05. The way the data were analyzed was exploratory. No adjustments for multiple tests were carried out. Since all patients treated for major trauma between March 2012 and February 2014 who had EQ-5D data at least at one of the three measured points of time were included, no sample size calculation was performed.

Frequency (n), percentage (%), mean (m), 95% confidence interval (95% CI), median (med), and interquartile range (IQR) were used in descriptive analyses. To compare the baseline characteristics of male and female patients, chi-square tests or Fisher’s exact tests were used for nominal data and U-tests were used for metric or ordinal data. Five EQ dimensions were compared using Fisher’s exact tests (no problem versus any problem)

Six, twelve, and twenty-four months after trauma within and between sex (interaction effect time × sex), repeated measures of HRQoL (EQ Index and EQ VAS) were assessed using mixed linear models (MLMs), maximum likelihood method, and unstructured repeated covariance type. To mitigate the effects of non-uniformly distributed confounders between the sexes, the MLMs incorporated variables that were presumed to have the potential to impact the outcomes. The linear mixed models included the following main effects: age (categorical: 16–24 vs. 25–39 vs. 40–64 vs. ≥65 years; main effect and interaction effects time × age and time × age × sex), RISC II (main effect), FCI (main effect), ASA physical status (main effect), and AIS for the body regions head, face, thorax, abdomen, extremities, and soft tissue. We chose age groups according to the guidelines on standard international age classifications by the Department of International and Social Affairs of the United Nations (New York, 1982) [29]. The MLMs used the full data set by filling in missing values with the most likely estimates. Thus, all patients, even those with missing quality-of-life values at specific time points, could be included for the analyses.

## 4. Results

### 4.1. Baseline Characteristics

The majority (57%) of the 416 patients who met the inclusion criteria (were treated in a level II trauma center (Table 1). A total of 72% male (300 patients) and 28% female patients (116 patients) participated in this study (Table 1). Most patients (43%) were between 40 and 64 years old and sustained high-velocity trauma in a motor vehicle collision (32%). A total of 91% of the patients were treated at the ICU, and 51% were intubated for a median time of 1 day (IQR = 0/5, n = 413). The median ISS was 22 (IQR = 17/29); the median RISC II was 1.6 (IQR = 1/615); the median FCI was 4 (IQR= 2/5); the median ASA was I (IQR = I/II); and the median GCS was 15 (IQR = 13/15).

### 4.2. Quality of Life

**Figure 2:**
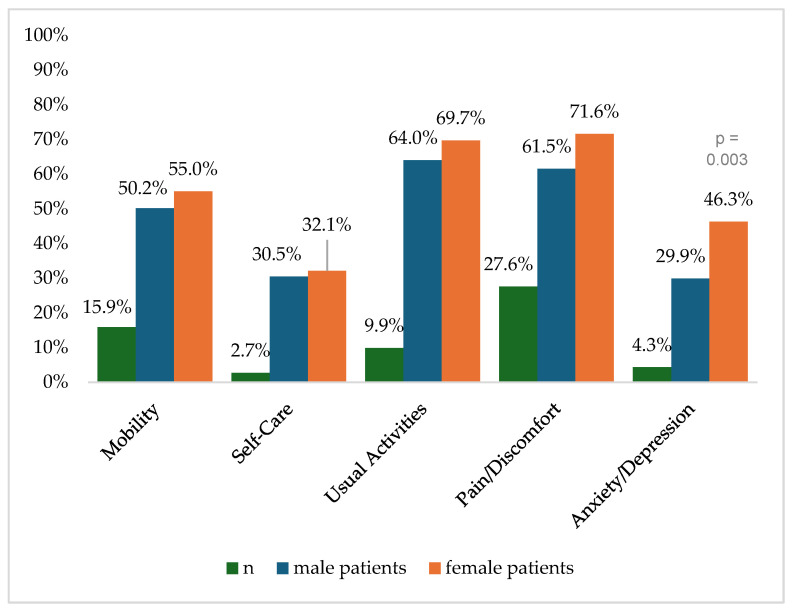
Reported problems in EQ-5D-3L dimension 6 months after trauma.

**Figure 3:**
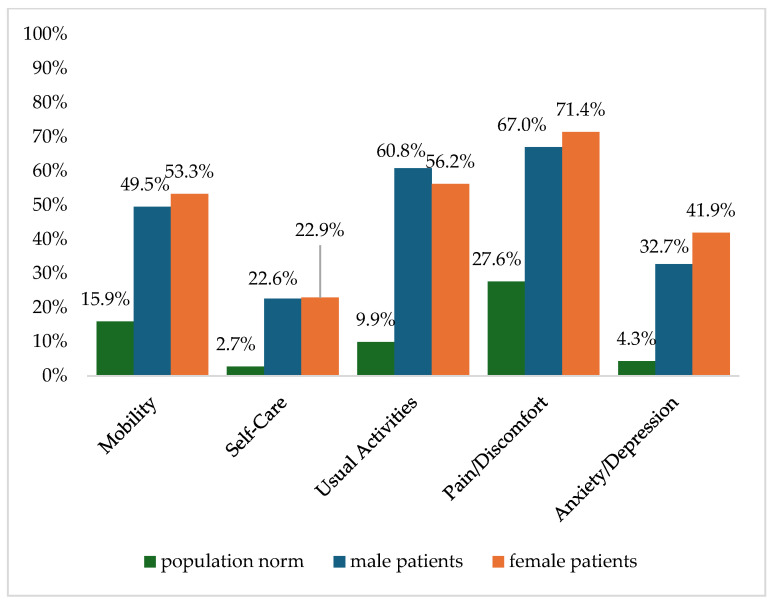
Reported problems in EQ-5D-3L dimension 12 months after trauma.

**Figure 4:**
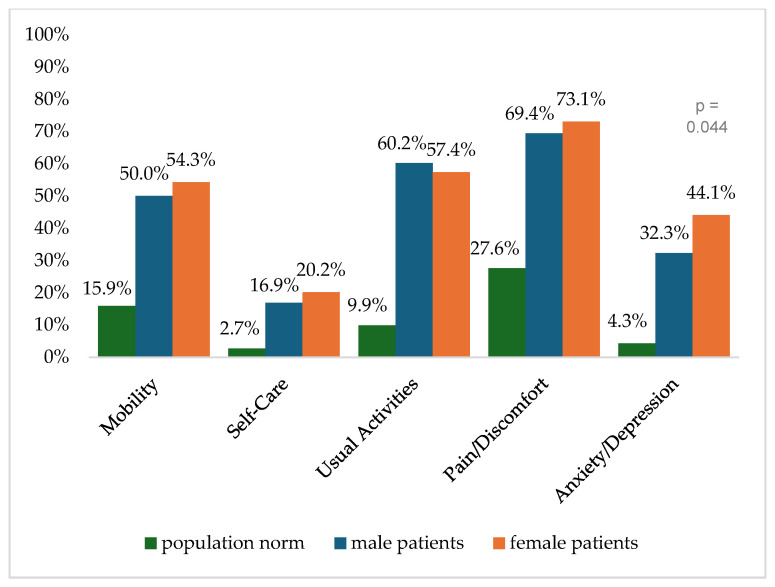
Reported problems in EQ-5D-3L dimension 24 months after trauma.

Figure 2 shows the reported problems of EQ-5D dimensions after 6 months. At 12 months post-trauma, female patients reported lower score values in EQ-5D for anxiety/depression (Figure 3). following severe injury. Long-term results for EQ-5D dimensions are presented in Figure 4. Compared to the German population norm, all patients reported more challenges in all five HRQoL dimensions.

Six months (*p* = 0.003) and twenty-four months (*p* = 0.044) after severe injury, female patients reported significantly higher rates of anxiety and depression than male patients.

### 4.3. Course of EQ Index and EQ VAS

The quality of life increased over time (Table 2). The EQ VAS increased from 6 months to 12 months after trauma (*p* < 0.001) and remained stable from 12 to 24 months after trauma (*p* = 0.645). In contrast, the EQ Index did not differ between 6 and 12 months after trauma (*p* = 0.146) but was significantly higher 24 months post-trauma than 6 months (*p* = 0.002) and 12 months post-trauma (*p* = 0.012).

In terms of the EQ Index and the EQ VAS, there was no significant difference between male and female patients (Table 3). The EQ VAS rose from 6 to 12 months after trauma (*p* < 0.001) and stayed constant from 6 to 24 months after trauma (*p* = 0.914) in male patients, while the EQ Index increased significantly from 6 to 24 months after trauma (*p* = 0.030). Within female patients, the EQ Index 24 months post-trauma was significantly higher than 6 months (*p* = 0.030) and 12 months (*p* = 0.047) after trauma. No further significant differences were found.

The analyses of the course of quality of life between age groups (Table 4) and the course of quality of life separated for age and sex (Table 5) showed that patients ≥65 years of age did not show any significant changes in the EQ Index and EQ VAS over time. Moreover, patients aged 16–24 years and 25–39 years showed no changes in the EQ Index over time. Significant changes were found for patients aged 40–64 in the EQ Index and the EQ VAS as well as for patients aged 16–24 and 25–39 in the EQ-VAS. These changes were similar; quality of life increased significantly from 6 to 12 months after trauma (*p*-values < 0.50) and was stable between 12 and 24 months after trauma. The most improvement is shown in patients between 16 and 39 years of age, especially female patients. Females over 65 years of age showed a low EQ-5D Index, which remained significantly lower than their male counterparts in their age group.

## 5. Discussion

This study evaluates different dimensions of health-related quality of life of female patients after severe trauma. After major trauma, most patients suffer from functional and personal consequences, often impacting their long-term quality of life, which often remains far below the population norm [30]. Since the overall mortality after severe trauma is diminishing, quality of life and other patient-reported outcomes after survival, as well as the identification of neglected patients who need special attention and care, are gaining importance. Research concerning health-related quality of life can reveal the prolonged impact of severe trauma [31]. The evaluation of quality of life requires a laborious longitudinal follow-up, and an understanding of influencing factors of trauma patients’ quality of life perceptions could lead to new strategies to improve patient outcomes and care. Regardless of age and sex, our results show a limited HRQoL in all patients in our cohort, particularly at 24 months. It is important to address these issues regardless of sex differences and their potential impact on resource allocation for trauma survivorship programs [3,32].

There have been mixed findings from several studies looking at how gender and sex affect morbidity and mortality following severe trauma. Multiple studies showed no difference in mortality and morbidity between the sexes [13]. Given the increasing number of female trauma patients, a more thorough examination of the impact of sex on outcome is imperative [33]. Prima vista, our results show no significant difference between male and female patients regarding both the EQ Index and the EQ VAS 6, 12, and 24 months after trauma. However, over all age groups, female patients reported significantly more problems in the subgroups of anxiety and depression than male patients. This is not unexpected since depression prevalence in general is higher in females, and severely injured patients are prone to develop depression and post-traumatic stress disorder (PTSD) [34]. Depression and PTSD are independent predictors of worse outcomes regardless of injury severity [35]. The role of the social construct of gender and its impact on the experience of trauma should not be underestimated. Female patients are known to be twice as likely to develop PTSD symptoms, depression, and physical anxiety as male patients, which could be attributed to higher levels of associated risk factors [36].

The sex differences in the psychological state post-trauma are not fully understood but need further investigation and treatment since they put female patients at risk for negative consequences regarding the outcome [37]. While the raw data suggest more problems per domain for females over the age of 65 years compared to men, the adjusted data (Table 4) show fairly similar results in VAS by 24 months. Whether these results are due to social support, similar coping mechanisms, and resilience should be further investigated in future studies.

In our study, we found that female patients between 16 and 39 years of age showed the most improvements in HRQoL. There are conflicting data on whether premenopausal females are more resistant to shock after severe injury [13]. Experimental studies suggest a high level of estrogen is protective after severe injury and hemorrhage [6,11,38]. Several studies showed that premenopausal female patients show a reduced mortality rate with significantly less multi-organ failure and sepsis compared to men of their age group [12,19]. Other studies could not detect any difference in mortality and severe complications in premenopausal female patients compared to male patients [7,8,12,14,18,19,20].

Concordant with existing literature on the topic, the analysis of the course of quality of life within the age group showed that patients ≥65 years of age did not show any significant changes in HRQoL over time, whereas patients <65 years of age showed significant improvement [39]. Our results reveal significantly worse outcomes for female patients over 65 years of age than for men of the same age. Their EQ-5D Index remained significantly lower over time than for their male counterparts. Even though, to our knowledge, this finding has not explicitly been reported for HRQoL in postmenopausal females before, there are several hints that elderly female patients are at risk for worse outcomes [16]. Prolonged hospital stays, less efficient rehabilitation, pre-existing illness, immunosuppression, misjudgment of injury severity, and enhanced mortality are documented and contribute to adverse outcomes in elderly females [9,40,41,42,43,44].

Since there is male dominance in injury frequency and severity, the impact of trauma on female patients might be masked and undervalued. Therefore, further research is warranted to address the needs of the potentially neglected group of elderly women in terms of both treatment and prevention [41]. Such research could provide valuable insights into their specific needs and preferences. Additionally, examining the benefits of support measures for elderly females and special training and sensibilization of personnel could ultimately enhance the quality of care and support provided [32].

Our study is limited by the number of cases. Since most injuries were caused by high-velocity collisions, our results might not be transferable to countries where penetrating injuries are more common. Due to the large multicenter design, the possibilities of clinical follow-up examinations are limited [45]. Great efforts have been taken to improve data with a documentation rate of major trauma patients of >95% [46].

The maximum likelihood estimates are used by the MLM to replace missing values. As a result, the analyses could include all patients, even those whose quality-of-life values were missing for selected time points.

In long-term research, non-participation is typically a significant problem. By comparing the baseline characteristics of participants and non-participants, we were able to reduce the effect, as there was no discernible difference between the subgroups. Selection bias can still be introduced by participation dissent.

We may not have considered all potential confounding variables. However, our goal was to obtain objective estimates of HRQoL means, considering all clinically relevant factors and encompassing all major trauma patients in a particular geographic area. The prospective multicenter registry-based design and quality of data are a robust framework. To validate our findings, more research on the assessment of HRQoL in older females after major trauma would be beneficial.

## 6. Conclusions

All patients showed limited HRQoL after major trauma. Overall, the group of patients younger than 39 showed the most improvement in HRQoL, particularly in females. Female patients reported significantly more anxiety and depression after major trauma than male patients. Females over 65 years of age showed worse HRQoL and the least improvement. Thus, further development and methodologically rigorous testing of ortho-geriatric initiatives, psychosocial support, and prevention measures are required to improve the care after major trauma, particularly for the female elderly.

## Figures and Tables

**Table 1:** Baseline characteristics of the study population.

		Male Patients n = 300		Female Patients n = 116		*p* Value
Age	16–24	79	(26.2%)	14	(12.2%)	<0.001
	25–39	52	(17.3%)	16	(13.9%)	
	40–64	125	(41.5%)	53	(46.1%)	
	≥65	45	(15.0%)	32	(27.8%)	
Level of trauma center facility	Level I	112	(37.2%)	45	(39.1%)	0.457
	Level II	171	(56.8%)	66	(57.4%)	
	Level III	18	(6.0%)	4	(3.5%)	
Intensive care unit	no	275	(91.4%)	105	(91.3%)	1.000
	yes	26	(8.6%)	10	(8.7%)	
Intensive care unit stay (in days)		5	(2/13)	4	(2/13)	0.731
ISS ^1^		22.0	(18.0/30.0)	20.0	(17.0/27.5)	0.462
	AIS head	2.0	(0/3.0)	2.0	(0/4.0)	0.347
	AIS face	0	(0/0)	0	(0/0)	0.398
	AIS thorax	3.0	(2.0/4.0)	3.0	(0/4.0)	0.668
	AIS abdomen	0	(0/2.0)	0	(0/2.0)	0.538
	AIS extremities	2.0	(0/3.0)	2.0	(0/2.5)	0.097
	AIS soft tissue	0	(0/1.0)	0	(0/1.0)	0.098
RISC II ^2^		1.2	(0.7/3.9)	6.3	(2.2/16.5)	0.689
FCI ^3^		4.0	(2.0/5.0)	5.0	(3.0/5.0)	0.485
ASA physical status ^4^		1.0	(1.0/1.0)	2.0	(2.0/3.0)	0.281
Type of injury	penetrating	10	(3.4%)	4	(3.5%)	1.000
	blunt	282	(96.6%)	109	(96.5%)	
Time accident to surgery (in hours)		3.0	(2.3/4.3)	2.9	(2.4/5.0)	0.670
In-hospital stay (in days) ^6^		15.5	(9.7/24.0)	17.4	(10.5/27.4)	0.135
Emergency surgery	yes	74	(26.5%)	34	(32.1%)	0.310
	no	205	(73.5%)	72	(67.9%)	
Mechanism of injury	car	81	(27.4%)	52	(45.6%)	<0.001
	motorcycle	77	(26.0%)	4	(3.5%)	
	bike	18	(6.1%)	12	(10.5%)	
	pedestrian	12	(4.1%)	10	(8.8%)	
	fall from great high	54	(18.2%)	8	(7.0%)	
	Low-level fall	28	(9.5%)	15	(13.2%)	
	hit	10	(3.4%)	4	(3.5%)	
	stabbing	1	(0.3%)	1	(0.9%)	
	other	10	(3.4%)	8	(7.0%)	

The data display the number of patients as a percentage of all patients for categorical variables, the median (IQR) for metric variables, and *p*-value for comparing patient sex groups. ^1^ ISS (Injury Severity Score) n = 300/n = 116; ^2^ RISC II (Revised Injury Severity Classification Score II) n = 275/n = 103; ^3^ FCI (Functional Capacity Index) n = 297/n = 116; ^4^ ASA physical status (American Society of Anesthesiologists score) n = 278/n = 112; ^6^ in-hospital stay: n = 68/n = 31.

**Table 2:** The course of quality-of-life data.

	6 Months Post-Trauma				12 Months Post-Trauma				24 Months Post-Trauma				*p*-Value
	n	m	95% CI		n	m	95% CI		n	m	95% CI		
EQ index	318	0.71	0.68	0.75	330	0.74	0.71	0.77	298	0.78	0.75	0.81	0.006
EQ VAS	325	62.0	59.1	64.8	331	68.7	66.1	71.2	301	69.3	66.6	72.0	<0.001

**Table 3:** The course of quality-of-life data separated for sex (^1^ *p* < 0.05).

	6 Months Post-Trauma				12 Months Post-Trauma				24 Months Post-Trauma				*p*-Value
	n	m	95% CI		n	m	95% CI		n	m	95% CI		
**EQ Index**													
male	226	0.72	0.68	0.76	239	0.71	0.78	0.74	219	0.77	0.74	0.80	0.050
female	92	0.71	0.65	0.78	91	0.68	0.79	0.73	79	0.79	0.73	0.85	0.070
*p* value ^1^	0.897				0.768				0.538				
**EQ VAS**													
male	233	62.6	59.6	65.6	240	67.6	64.9	70.2	221	67.4	64.7	70.1	<0.001
female	92	61.3	56.4	66.2	91	69.8	65.4	74.2	80	71.1	66.4	75.8	<0.001
*p* value ^1^	0.658				0.393				0.183				

**Table 4:** The course of quality-of-life data separated for age (^1^ *p* < 0.05).

	6 Months Post-Trauma				12 Months Post Trauma				24 Months Post-Trauma				*p* Value
	n	m	95% CI		n	m	95% CI		n	m	95% CI		
**EQ Index**													
16–24 years	73	0.79	0.70	0.87	73	0.82	0.75	0.90	69	0.86	0.78	0.94	0.319
25–39 years	45	0.73	0.64	0.82	53	0.75	0.68	0.83	49	0.81	0.74	0.88	0.174
40–64 years	145	0.66	0.61	0.71	149	0.71	0.66	0.75	136	0.74	0.69	0.78	0.019
≥65 years	55	0.68	0.60	0.76	55	0.68	0.61	0.75	44	0.72	0.64	0.80	0.546
*p* value ^1^	0.068				0.038				0.017				
**EQ VAS**													
16–24 years	77	68.1	61.6	74.6	73	78.1	72.1	84.1	71	78.3	72.1	84.6	<0.001
25–39 years	48	62.3	55.7	69.0	54	67.9	62.1	73.7	49	70.8	64.9	76.7	0.024
40–64 years	145	58.5	54.8	62.3	149	66.6	63.2	69.9	136	65.0	61.5	68.4	<0.001
≥65 years	55	58.9	52.7	65.0	55	62.2	56.6	67.7	45	63.0	56.8	69.3	0.279
*p*-value ^1^	0.080				0.002				0.001				

**Table 5:** The course of quality-of-life data separated for age and sex.

	6 Months Post-Trauma				12 Months Post-Trauma				24 Months Post-Trauma			
	n	m	95% CI		n	m	95% CI		n	m	95% CI	
**EQ Index** **male patients**												
16–24 years	60	0.80	0.73	0.87	62	0.83	0.77	0.89	59	0.84	0.78	0.90
25–39 years	31	0.68	0.58	0.77	39	0.75	0.68	0.83	35	0.75	0.67	0.83
40–64 years	100	0.65	0.60	0.71	104	0.69	0.64	0.74	93	0.74	0.69	0.78
≥65 years	34	0.74	0.64	0.84	34	0.70	0.62	0.79	32	0.75	0.67	0.84
**EQ Index** **female patients**												
16–24 years	13	0.78	0.62	0.93	11	0.81	0.67	0.96	10	0.88	0.74	10.02
25–39 years	13	0.78	0.63	0.93	14	0.75	0.62	0.87	14	0.87	0.75	0.99
40–64 years	45	0.67	0.59	0.75	45	0.72	0.65	0.79	43	0.74	0.67	0.81
≥65 years	21	0.62	0.50	0.74	21	0.66	0.55	0.76	12	0.68	0.56	0.81
**EQ VAS** **male patients**												
16–24 years	64	67.7	62.4	73.0	62	77.7	72.9	82.5	61	78.3	73.4	83.2
25–39 years	35	57.6	50.7	64.5	40	65.8	59.8	71.8	35	64.4	58.1	70.6
40–64 years	100	59.7	55.5	63.8	104	63.2	59.6	66.9	93	64.9	61.1	68.7
≥65 years	34	65.4	58.0	72.9	34	63.5	56.8	70.3	32	62.1	55.2	69.1
**EQ VAS** **female patients**												
16–24 years	13	68.5	56.8	80.2	11	78.6	67.7	89.5	10	78.4	66.9	89.9
25–39 years	13	67.1	55.7	78.5	14	70.0	60.1	79.9	14	77.2	67.3	87.2
40–64 years	45	57.4	51.1	63.6	45	69.9	64.3	75.4	43	65.0	59.3	70.7
≥65 years	21	52.3	43.1	61.5	21	60.8	52.6	69.0	13	63.9	54.1	73.7

## Data Availability

The data are available upon reasonable request.
